# A Community-Engaged, Mixed-Methods Approach to Prioritizing Needs in a Statewide Assessment of Community Cancer Needs

**DOI:** 10.5888/pcd21.240183

**Published:** 2024-12-26

**Authors:** Jessica R. Thompson, Todd Burus, Caree McAfee, Christine Stroebel, Madeline Brown, Keeghan Francis, Melinda Rogers, Jennifer Knight, Elaine Russell, Connie Sorrell, Elizabeth Westbrook, Pamela C. Hull

**Affiliations:** 1 Community Impact Office, Markey Cancer Center, University of Kentucky, Lexington; 2 Department of Health Policy and Administration, College of Health and Human Development, The Pennsylvania State University, University Park; 3 Kentucky Cancer Program, Community Impact Office, Markey Cancer Center, University of Kentucky, Lexington; 4 Department of Health Management and Policy, College of Public Health, University of Kentucky, Lexington; 5 Kentucky Cancer Consortium, Community Impact Office, Markey Cancer Center, University of Kentucky, Lexington; 6 Kentucky Cancer Program, University of Louisville, Louisville, Kentucky; 7 Department of Behavioral Science, College of Medicine, University of Kentucky, Lexington

## Abstract

**Introduction:**

Kentucky has the highest all-site cancer incidence and death rate in the US. In 2021, the University of Kentucky Markey Cancer Center convened a steering committee to conduct a statewide community cancer needs assessment (CNA). The goal of the final CNA phase was to gather community input on prioritizing Kentucky’s cancer-related needs and ways to address them.

**Methods:**

In 2021, we recruited 162 people to participate in online concept mapping, a participatory mixed method, to explore connections and identify priority areas. Fifty-one community members and 111 organizational partners participated in survey-based activities to prioritize 80 items representing key CNA findings and discussion groups to explore key focus areas and strategies for Kentucky communities.

**Results:**

Concept maps display perceived similarity of the 80 items and a 6-cluster solution. High-priority focus areas included lung cancer screening, smoking, human papillomavirus (HPV) vaccination, and disparities driven by social determinants among rural, Appalachian, Black, and Hispanic residents. High-priority strategies to address needs included expanding health communication on risks, screening guidelines, and insurance benefits; patient navigation; accessible, culturally appropriate treatment information and self-efficacy in treatment decisions; access to care through financial assistance, mobile clinics, and at-home screening; and patient–provider trust and communication.

**Conclusion:**

Our findings indicate the utility of the concept mapping process to facilitate the prioritization of wide-ranging catchment area needs and ways to address them. Moving forward, the prioritized focus areas and strategies can inform Kentucky’s new state cancer plan and future research to reduce the state’s cancer burden and disparities.

**Summary**

**What is already known on this topic?**

Concept mapping, a participatory approach, is used to assess community health needs. With the nation’s highest all-site cancer incidence and mortality, Kentucky residents have a wide range of cancer needs.

**What is added by this report?**

Through a cancer center–community collaboration, we used a novel online concept mapping approach to capture statewide perspectives from organizational partners and community members to prioritize cancer-related needs in Kentucky.

**What are the implications for public health practice?**

Our findings indicate the utility of concept mapping to facilitate the prioritization of wide-ranging catchment area needs. The prioritized areas can be used to guide the state’s cancer plan and future research to reduce cancer burden.

## Introduction

Concept mapping is a participatory method used to generate consensus on a specific topic (1,2). This mixed method captures in-depth experiences through qualitative data with the ability to structure and prioritize findings for new agendas. Concept mapping has been used to assess community health and cancer-related needs (3,4), including breast cancer screening (5), rural patients with head and neck cancers (6), prostate cancer treatment decisions (7), navigation experiences of breast cancer patients (8), human papillomavirus (HPV) vaccination strategies (9), and cancer survivor needs (10). Over time, online concept mapping tools have added user-friendly elements for direct participation in a web-based platform (11). The need for online options for community-based or qualitative data collection tools increased during the COVID-19 pandemic (12–14). By conducting concept mapping online, the participant pool widens to include broad geographic areas and people who face challenges, such as transportation or childcare, in attending in-person sessions (12).

Promoted by accreditation boards and the Patient Protection and Affordable Care Act, the use of needs assessments among public health agencies, nonprofit hospitals, and state cancer coalitions has grown in recent decades. For example, National Cancer Institute-designated cancer centers are required to regularly assess catchment area needs to develop priorities for health care, research, and cancer control activities (15,16). The University of Kentucky Markey Cancer Center’s (UKMCC’s) catchment area is the state of Kentucky. With the country’s highest all-site cancer incidence and mortality (17), Kentucky residents have a wide range of cancer-related needs across the care continuum from risk reduction to treatment follow-up. The UKMCC Community Outreach and Engagement team convened a steering committee to collaborate on a new cancer needs assessment (CNA). We leveraged online concept mapping as a unique opportunity to capture statewide perspectives from partner organizations and community members to prioritize needs and ways to address them.

To our knowledge, this is the first study by a cancer center–community collaboration to use concept mapping to prioritize cancer needs and strategies. We aimed to 1) identify the range of perceived barriers and facilitators for cancer risk reduction, screening, treatment, and survivorship among Kentuckians; 2) assess the relationships among identified barriers and facilitators based on perceived importance and feasibility to address; and 3) identify data- and community-driven priorities to improve cancer control activities in the state.

## Methods

### Kentucky CNA

Kentucky maintains an extensive partnership infrastructure to improve cancer prevention and outcomes. Created in 2002 and guided by the UKMCC Community Outreach and Engagement team, the Kentucky Cancer Consortium (KCC) is the state’s comprehensive cancer control coalition, which develops and implements Kentucky’s cancer plan. The Kentucky Cancer Program (KCP) was founded in 1982 as the state’s cancer prevention and control program. KCP-West is led by the University of Louisville, and KCP-East is directed by the University of Kentucky. KCP staff organize and implement evidence-based programs with various local and regional partners.

In 2021, the UKMCC Community Outreach and Engagement team convened a steering committee to drive creation of a new CNA for Kentucky, including statewide organizational partners, clinicians, academics, national foundation representatives, and others engaged in cancer control. Initially, quantitative data used in such assessments were gathered (18–20) to establish current cancer trends and risk factors (16). Additionally, qualitative data, through a series of community focus groups, were collected to understand cancer experiences, perceptions, and needs of Kentuckians. This statewide CNA resulted in a wide range of potential focus areas requiring prioritization for action.

### Research design

**Figure 1:**
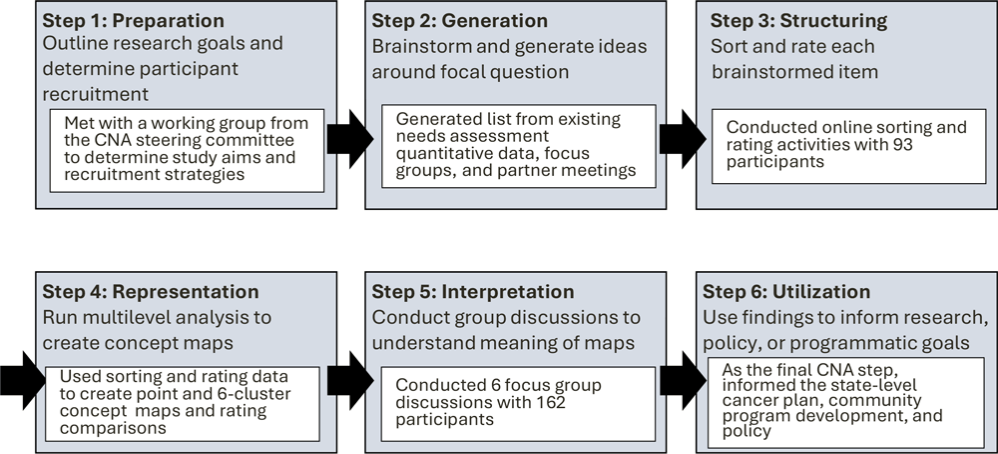
Six steps of concept mapping and project activities. The general steps are indicated at the top of each box, and each inset describes the activity conducted during the project, a cancer needs assessment (CNA) in Kentucky.

This study used a mixed-methods, observational design through concept mapping (1,2,4). The concept mapping process involves sequential activities (Figure 1): preparation (Step 1), generation (Step 2), structuring (Step 3), representation (Step 4), interpretation (Step 5), and utilization (Step 6) (2). Data collection typically occurs at 3 points: when brainstorming a list of responses to a focal question (Step 2), when structuring the listed ideas through sorting and rating (Step 3), and when interpreting the generated concept maps and patterns through qualitative group discussions (Step 5). 

### Study populations and recruitment

Beginning in September 2021 (Step 1: Preparation), we recruited 2 groups: 1) adult community members who are nonhealth professionals and Kentucky residents and 2) staff of statewide and community-based partner organizations in Kentucky. Due to an active COVID-19 pandemic wave, all recruitment was performed via email. To recruit community members, we contacted 109 people previously screened for the CNA focus groups who had consented to be recontacted for future studies. We initially recruited these people through flyers distributed through the KCP and KCC partnership networks as well as ResearchMatch (21), a national registry of potential research participants. The previously recruited participants had an average age of 49 years, with 35% from rural communities and 31% who identified as a racial or ethnic group other than non-Hispanic White. To recruit statewide and community-based organizational representatives, we invited 186 people from KCC and KCP networks, including health departments, foundations, nonprofit organizations, advocacy groups, health systems, health insurance companies, and educational organizations.

The study team contacted prospective participants via an email invitation, which provided a study cover letter with consent language and a link to the online concept mapping platform. The community member participants received up to $60 in e-gift cards for participation ($30 for the online activities and $30 for the group discussion). The organizational partner representatives participated in their professional roles. All procedures were approved by the University of Kentucky Institutional Review Board as an expedited study with a waiver of consent documentation (no. 73420).

### Data collection and analysis

From September through December 2021, we conducted online concept mapping activities using GroupWisdom (11) and Zoom video conferencing (Zoom Video Communications, Inc). Participants could choose to take part in a single activity or all data collection activities. If participants expressed concern about technology during the eligibility screening process, we offered one-on-one sessions for guidance. Additionally, we included study contact information at every concept mapping step to allow participants to raise questions or concerns. We provided regular reminders to maximize participation in each step.

**Brainstorming (Step 2: Generation)** *.* The authors, a working group of steering committee members, collated data from CNA quantitative data, CNA focus group themes, and common topics raised in KCC and KCP meetings identified through minutes and action items. The use of 3 sources allowed us to triangulate items, to remove duplicates, and to synthesize these items into a single list for use in the subsequent concept mapping activities. The final 80-item list (Appendix) includes topics ranging from health indicators to community and health care obstacles. All 80 items were developed in response to a focal question: “What things, good or bad, impact cancer prevention risk reduction, screening, treatment, or survivorship in Kentucky communities?” We loaded the final list of items into the GroupWisdom online concept mapping platform.

**Sorting and rating (Step 3: Structuring).** Next, we invited participants to perform sorting and rating activities in the online GroupWisdom platform, which provides detailed instructions to walk participants through each assigned activity. We first asked participants to sort the 80 items into piles they perceived belonged together and to assign a thematic name to each pile. We then asked participants to rate each item on 2 Likert-type scales: 1) How important is this item for Kentucky communities? and 2) How easy would it be to address this item in Kentucky communities? The response choices ranged from 1 (not at all important/not at all easy) to 5 (extremely important/extremely easy). We collected demographic information during this step (age, race, ethnicity, educational attainment, health insurance status, gender identity, LGBTQ+ [lesbian, gay, bisexual, transgender, queer] identity, and family history of cancer).

**Quantitative analyses (Step 4: Representation).** We used nonmetric multidimensional scaling with the sorting data to create a spatial point map, which uses the relative distances between items to reflect perceived similarities, and hierarchical cluster analysis to depict group consensus on thematic overarching categories in a cluster map; we combined these into a single map (2). Additionally, we examined comparisons for average cluster ratings across the rating scales, including correlational values (*r*). For the highly rated clusters, we created go-zone plots, which use bivariate comparisons to demonstrate which items are highly rated across both scales.

**Discussion Groups (Step 5: Interpretation).** Finally, in December 2021, we showed the combined point-and-cluster map, rating comparisons, and go-zone plots to participants for interpretation. These sessions followed the structure of a qualitative focus group, where participants reacted to the maps in a semistructured, guided discussion. Overall, we conducted 6 interpretation sessions: 3 with community members and 3 with organizational partners. The community member participants developed the cluster names in breakout rooms based on their perceptions of commonality among the items in each cluster, and a large group discussion ensured consensus among participants. In the organizational partner groups, we discussed prioritization of focus areas for future work in cancer control. During all interpretation sessions, participants were prompted to discuss the clusters most highly rated across both rating scales. We paid special attention to exploring strategies for addressing identified barriers and challenges and observing differences by participant type and geographic area in Kentucky. We recorded the discussion groups and transcribed the recordings, which were used to identify representative quotes.

## Results

Overall, 162 people participated in this study. Ninety-three of these people participated in the online sorting and rating activities and answered the demographic questions (Table 1). These online activity participants had a mean age of 50.0 years and were majority non-Hispanic White (82.8%). Approximately 90% of participants identified that they or an immediate family member had a history of cancer, and participants lived in 44 different counties, including 39.3% in rural and 22.5% in Appalachian counties. Our community member participants had greater diversity, including race, ethnicity, education, and insurance type, than the organizational partners.

**Table 1:** Demographic Characteristics of Participants Who Completed Sorting and Rating Activities in the Concept Mapping Process Conducted as Part of a Statewide Assessment of Community Cancer Needs, Kentucky, 2021^a^

Characteristic	Community members (n = 51)	Organizational partners (n = 42)	All (N = 93)
**Age, mean (SD), y**	49.9 (13.5)	50.1 (13.6)	50.0 (13.5)
**Race and ethnicity^b^**			
American Indian or Alaska Native	2 (3.9)	1 (2.4)	3 (3.2)
Asian or Asian American	3 (5.9)	0	3 (3.2)
Black or African American	11 (21.6)	4 (9.5)	15 (16.1)
Hispanic, Latin American, or Spanish origin	1 (2.0)	0	1 (1.1)
White	39 (76.5)	38 (90.5)	77 (82.8)
**Education**			
Completed high school or GED	4 (7.8)	2 (4.8)	6 (6.4)
Some college or vocational school	10 (19.6)	4 (9.5)	14 (15.0)
Bachelor’s degree or higher	37 (72.6)	36 (85.7)	73 (78.5)
**Health insurance^b^**			
Employer, military, or private	29 (56.9)	37 (88.1)	66 (71.0)
Medicaid	10 (19.6)	1 (2.4)	11 (11.8)
Medicare	14 (27.4)	3 (7.1)	17 (18.3)
Kentucky health insurance marketplace	1 (2.0)	1 (2.4)	2 (2.2)
**Gender identity**			
Woman	39 (76.5)	35 (83.3)	74 (79.6)
Man	11 (21.6)	5 (11.9)	16 (17.2)
Non-binary or Genderqueer	1 (2.0)	2 (4.8)	3 (3.2)
**Identified as LGBTQ+**			
Yes	5 (9.8)	2 (4.8)	7 (7.5)
No	46 (90.2)	39 (92.9)	85 (91.4)
Prefer not to answer	0	1 (2.4)	1 (1.1)
**Family (including participant) history of cancer**			
Yes	45 (88.2)	39 (92.9)	84 (90.3)
No or not to my knowledge	6 (11.8)	3 (7.1)	9 (9.7)

Abbreviations: GED, General Educational Development; LGBTQ+, lesbian, gay, bisexual, transgender, queer.

a All values are number (percentage) unless otherwise indicated. Percentages may not add to 100 because of rounding.

b Responses not mutually exclusive.

### Cluster maps and names

**Figure 2:**
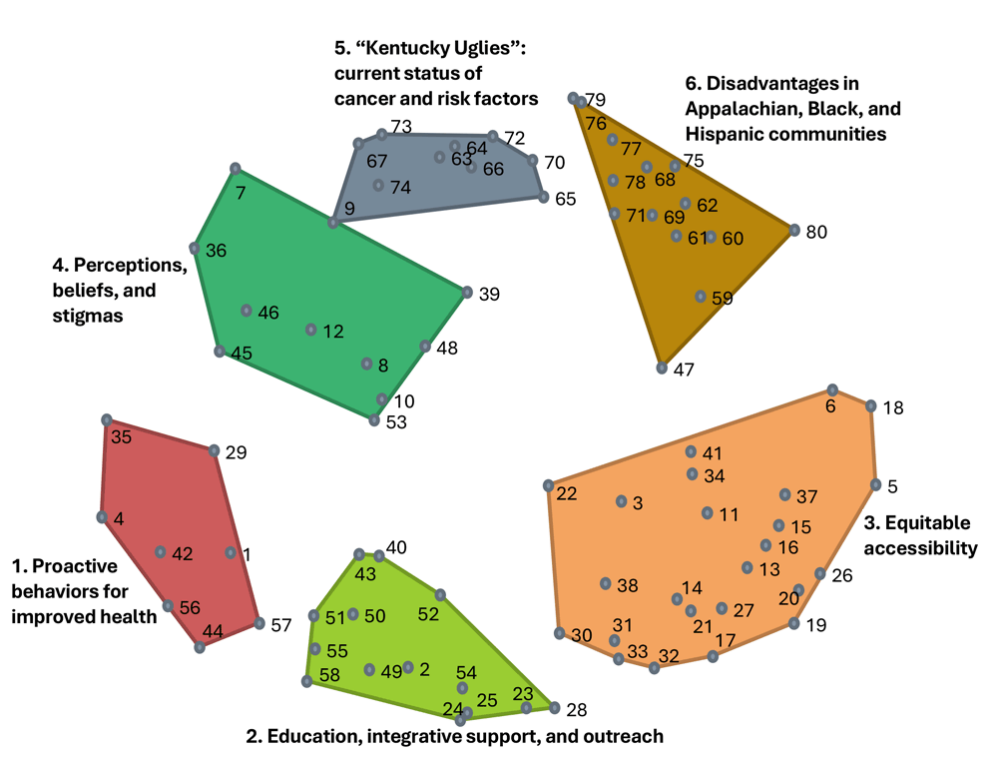
Combined point-and-cluster maps resulting from sorting and rating data. Cluster names were developed by participants in a cancer needs assessment in Kentucky. Relative distances between items reflect perceived similarities.

The best cluster solution resulted in a 6-cluster map, which grouped the 80 items into 6 thematic areas (Figure 2). The cluster names are “Proactive behaviors for improved health” (Cluster 1); “Education, integrative support, and outreach” (Cluster 2); “Equitable accessibility” (Cluster 3); “Perceptions, beliefs, and stigmas” (Cluster 4); “’Kentucky Uglies’: current status of cancer and risk factors” (Cluster 5); and “Disadvantages in Appalachian, Black, and Hispanic communities” (Cluster 6).

### Topics of focus

Topic areas for partnerships and organizations to address were derived from the cancer risk factors, outcomes, and issues of health equity found in Clusters 5 and 6.

Across Kentucky, items 72 (“About 1 in 4 adults are current smokers, with higher rates in Appalachia [second-highest rate in the US]”) and 62 (“Lung cancer screening rates are low [even worse in the Black community]”) rose to the top among community partners. As one participant described:

I really like 62 [low lung cancer screening rates] and 72 [high smoking rates], but I would put 62 first because of how difficult it is to try to get people to stop smoking. So at least, if we could move those people toward lung cancer screening, but the thing is, we have to educate the community on who is eligible for lung cancer screening, what it entails, and all of that, but I think that’s a good start.

Participants also identified item 69 (“More Black Kentuckians die from certain cancers than White Kentuckians [examples: uterine, prostate, stomach, myeloma]”) and described the need for continued work addressing disparities in Kentucky’s Black population. For example, prostate cancer:

One of the things we have continued to promote is prostate screening among African Americans. We have not stopped doing that. We have been doing that for 30 years and as recently as the state fair this year. So, that’s going to remain on our radar because we have funders who want to [do] something and a community who understands there’s a need, so that’s something we’ll continue to do.

Item 65 (“Cancers related to HPV are higher than the US [some of the worst rates in cervical and head/neck cancers]”) also regularly arose in conversations, with participants identifying it as a realistic goal. One participant describes: “I think 65 could be really good because that’s something we can educate how to prevent and then to really promote the vaccine, and that’s one that really is attainable.” Finally, items 75 (“Higher levels of poverty among rural, Appalachian, Black, and Hispanic Kentuckians than the US”) and 70 (“Higher cancer death rates in counties with lower education versus higher education”) commonly emerged, indicating a continued importance for addressing social and economic determinants of health in cancer services.

Additional items were identified by participant type (community members and organizational partners), such as environmental exposures, breast/cervical cancer screening, tobacco use, and physical activity, which may be of interest for the development of cancer services. Likewise, a few items uniquely rose to the top by location of participants in western Kentucky or eastern Kentucky, such as obesity and colorectal screening (Table 2).

**Table 2:** Additional Identified Focus Areas and Strategies by Participant Type and Region in Kentucky^a^

Area or strategy	Participant type		Region	
	Community members	Organizational partners	Western Kentucky	Eastern Kentucky
**Focus area**	59. Breast cancer screening rates are similar to rates in the US but still need improvement 61. Lower rates of cervical cancer screening in rural areas and Appalachia 67. Some cancers linked to environment exposures are more common than in the US overall (examples: lung, kidney, melanoma, leukemia, bladder)	63. Cancers related to tobacco are higher than in the US overall (some of the worst rates of lung, head/neck, kidney, and bladder cancers) 74. 1 in 3 adults fail to get any physical activity outside of work (third worst in the US)	64. Cancers related to obesity are higher than in the US overall (some of the worst rates for colorectal, pancreatic, and brain cancers) 73. 1 in 5 youth and 2 in 5 adults are obese (among the highest rates in the US)	60. Lower colorectal cancer screening rates in Appalachia 74. 1 in 3 adults fail to get any physical activity outside of work (third worst in the US)
**Strategy**	**Cluster 2: Education, integrative support, and outreach**			
	24. Financial support for cancer treatment (examples: grants, foundation assistance, financial advisor)	55. Partnering with community organizations to share health information (examples: schools, faith-based, employers, local leaders)	55. Partnering with community organizations to share health information (examples: schools, faith-based, employers, local leaders) 58. Use of multiple media sources for health information (examples: mail, flyers, advertisements, websites, social media)	2. Health promotion programs by local organizations (examples: HPV vaccination, nutrition, tobacco cessation)
	**Cluster 3: Equitable accessibility**			
	27. Access to hospice or comfort care	11. Quality or trust of local health care facilities 41. Health information at low reading levels	22. Access to affordable nicotine replacement products (examples: patches, gum, lozenges) 27. Access to hospice or comfort care	3. Access to places with affordable healthy foods (examples: grocery stores, farmers markets) 38. Bilingual staff or interpreters 41. Health information at low reading levels

a Numbers refer to identification numbers for items identified in concept mapping (Appendix).

Overall, the high levels of commonalities among discussion groups suggest a continued focus on 1) improving rates of smoking and tobacco-related cancers (eg, lung cancer), 2) addressing cancer disparities in Black and Hispanic Kentuckians and in rural and Appalachian communities, 3) understanding the role of social determinants of health (eg, poverty, education), and 4) continuing to expand HPV vaccination and cervical cancer screening.

### Strategies for future cancer care efforts

**Figure 3:**
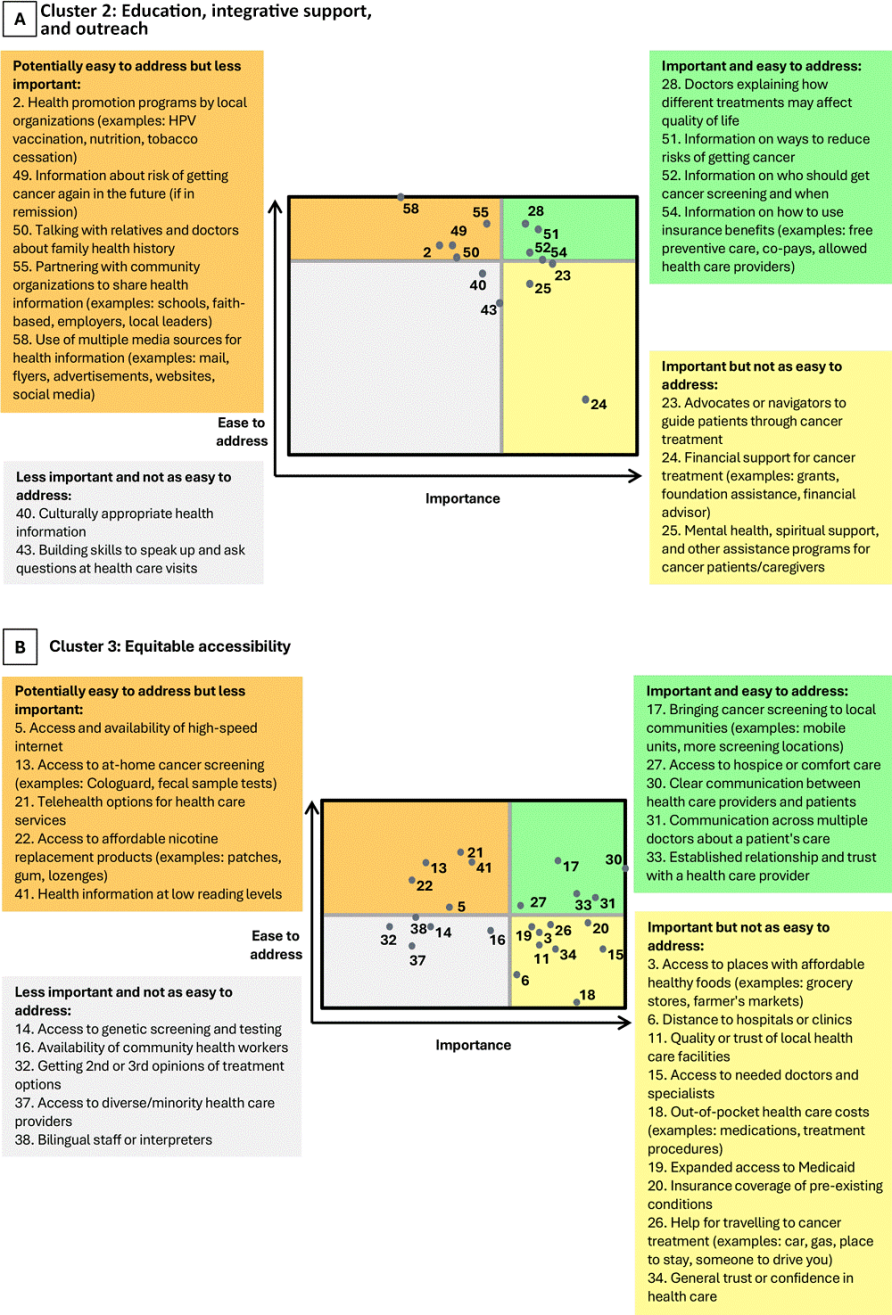
Go-zone plots for A) Cluster 2 (Education, integrative support, and outreach) and B) Cluster 3 (Equitable accessibility). Plots were used to demonstrate items highly rated across scales measuring responses to 2 questions: 1) How important is this item for Kentucky communities? and 2) How easy would it be to address this item in Kentucky communities? Quadrants are sized according to average item ratings. Items with above-average ratings are toward the top (ease to address) or right (importance). Items with below-average ratings are toward the bottom (ease to address) or left (importance).

The clusters most highly rated across both rating scales (importance and ease) were “Education, integrative support, and outreach” (Cluster 2) and “Equitable accessibility” (Cluster 3). The go-zone plots for these clusters (Figure 3) have a moderate and weak negative correlation (*r* = −0.58 for Cluster 2 and *r* = −0.16 for Cluster 3), indicating a diversity of thought about the importance and ease across the items in each cluster.

In Cluster 2, several strategies were raised by both community members and organizational partners, including health communication, particularly around screening information; treatment navigation, including the need for advocates and navigators; and building supports to provide accessible treatment information. For Cluster 3, participants commonly identified items in 2 major categories: access to care, such as ways to reach patients where they are and provide needed supports, and patient–provider trust and/or communication. Participants also highly rated several items as important but not necessarily easy to address, largely around access, insurance, and cost of care. Access to hospice and/or comfort care, partnering with local organizations, quality of local health care facilities, literacy levels, use of media sources, affordable nicotine replacement products, affordable healthy foods, and bilingual staff were strategy-related items uniquely identified by participant type or location (Table 2).

## Discussion

Our findings provide evidence for including concept mapping in needs assessments performed by public health agencies, nonprofit hospitals, and state cancer coalitions as well as catchment area assessments conducted by NCI-designated cancer centers. Previous publications on catchment area assessments largely focused on quantitative analyses of secondary data or community-based surveys with few using qualitative approaches (22–25). The inclusion of qualitative data in catchment area assessments captures participant perspectives that may not be reflected in quantitative trends, particularly among underresourced communities (ie, those with high poverty levels, geographic isolation, and/or reduced health care access) (26). Concept mapping adds further depth by generating consensus on an array of needs when resources may dictate the selection of specific action steps.

Concept mapping provides a unique opportunity to capture perspectives from both community members and organizational partners as well as the ability to make comparisons between these groups. Previous concept mapping studies made such comparisons to identify gaps in knowledge or discrepancy of views (3,5,8). In our study, the community member and organizational partner participants largely agreed on the item and cluster ratings for importance and ease to address, which allowed us to show the rating comparisons combined by participant type (eg, Clusters 2 and 3 were the most highly rated for all participants). Additionally, concept mapping, through its combination of individual quantitative activities with qualitative group discussions, allows for the inclusion of all voices, particularly those who may feel less comfortable contributing in a group setting. By including community members and organizational partners in an active way, we gain confidence that our findings will be useful and valid, both for the partners who will be instrumental in developing and implementing cancer risk reduction and control programs and directly for the community.

As with previous applications of concept mapping in health needs assessments (3,4), we found that participants rated all 80 items as important, indicating that community members and organizational partners alike felt that all the cancer needs identified in the CNA required attention. Likewise, our participants recognized the need for continued efforts in addressing the role of social and structural inequities in health outcomes (3). Our results also support previous findings in cancer-related concept mapping projects. For example, various individual (eg, psychosocial factors, financial impacts), social (eg, navigation of personal relationships, social supports [10]), and health care-related factors (eg, desire for health information from providers, access to services (5), empathetic and compassionate communication from providers, participation in one’s care decision-making, ways to address fears and anxiety in diagnoses [8]) all emerged in our project. These consistent findings highlight the continued importance of developing strategies related to health communication, compassionate and culturally appropriate ways of sharing information, and the development of continued supports, both financial and social, in cancer care.

Topically, our findings suggest the prioritization of strategies centered on lung cancer risk reduction and screening, including a focus on high smoking rates (Step 6: Utilization). Essential to addressing many forms of cancer, issues of health equity should continue to be a priority, including factors affecting Black and Hispanic Kentuckians and those in rural and low-income communities. Community- and partner-driven strategies to affect these areas include a continued focus on health communication strategies, supports for treatment navigation, ways to overcome barriers to access to care, and methods for increasing trust in patient–provider relationships. Service providers and health care professionals can build on these strategies, which are being included as part of Kentucky’s new statewide cancer plan. Since the CNA, the state passed legislation to establish a Kentucky Lung Cancer Screening Program; the bill was signed in July 2022. The findings from this study will inform the activities of this program along with other community cancer risk reduction and control research and services to reduce cancer incidence and mortality in Kentucky.

### Limitations

We acknowledge several study limitations. First, because we recruited participants remotely, we may have reached participants with better access to the internet and technology, which may reflect higher levels of geographic access to internet service providers and/or income. However, in previous work, we found that less than 5% of potential participants expressed concerns about online qualitative data collection, and the GroupWisdom platform provides supports for people participating on mobile devices, increasing the likelihood of participation among those without a computer or broadband internet. We also sought to mitigate any digital literacy issues through one-on-one assistance for those who requested guidance and the step-by-step walk-through for each activity provided by GroupWisdom. Additionally, the use of the GroupWisdom platform does require the purchase of a license, which may be cost prohibitive to small community organizations, although nonprofit organizations do qualify for a discount. We observed high participation levels among organizational partner participants who identified as non-Hispanic White, have higher educational attainment, have employer or private insurance, and identify as women, which may reflect the demographic characteristics of the health professions but may not represent the opinions of more diverse populations. Our community member participants consisted of more diverse people and do reflect the overall diversity of Kentucky residents; the 2020 US Census estimates indicate that 38.2% live in rural areas, 26.7% live in Appalachia, and 82.4% identify as non-Hispanic White (27). Although our goal was to broadly capture perspectives across the state, future studies should seek to understand additional viewpoints through the over-recruitment of various racial and ethnic groups, gender identities, and income levels. Likewise, our participants lived in 44 of the 120 Kentucky counties, but their views may not reflect the entire state. Finally, this sample is large for typical concept mapping studies, where sample size is not meant to be generalizable but rather to reach group consensus. As such, our findings are fairly robust and allow for the novel identification of cancer need prioritization in Kentucky.

### Conclusion

Overall, our findings indicate the utility of concept mapping for prioritizing wide-ranging catchment area needs uncovered in a CNA. We condensed 80 items into 6 thematic cluster areas for future exploration. Within these clusters, we identified concrete topics for future cancer prevention and control activities, including lung cancer screening, tobacco cessation treatment, and issues of health equity. We also identified community-driven action strategies in Kentucky, such as continuing to improve health communication, patient navigation, access to care, and culturally appropriate health information. As described elsewhere (28), these results provided guidance for overall CNA priorities, including a focus on lung cancer screening, tobacco cessation, and social determinants of health that drive disparities for Black, Hispanic, Appalachian, and rural Kentucky residents. These findings better position the UKMCC Community Outreach and Engagement team and steering committee members to address Kentucky’s cancer needs and reduce the state’s high cancer incidence and death rates.

## Appendix List of Final Concept Mapping Items Sorted by Cluster

**Table:**

Cluster	Item identification no.	Item
1. Proactive behaviors for improved health	1	Access to places to be active or exercise (examples: parks, sidewalks, gyms, recreation centers)
	4	Smoke-free policies for secondhand smoke exposure
	29	Side effects related to cancer treatment (examples: “chemo brain,” nutritional needs, gender/sexual health, pain)
	35	Health habits formed as children
	42	Building skills for healthy behaviors (examples: physical activity, sleep, healthy eating)
	44	Stress management and healthy coping skills
	56	Employer supports for healthy lifestyle choices
	57	Testimonials of personal cancer experiences
2. Education, integrative support, and outreach	2	Health promotion programs by local organizations (examples: HPV vaccination, nutrition, tobacco cessation)
	23	Advocates or navigators to guide patients through cancer treatment
	24	Financial support for cancer treatment (examples: grants, foundation assistance, financial advisor)
	25	Mental health, spiritual support, and other assistance programs for cancer patients/caregivers
	28	Doctors explaining how different treatments may affect quality of life
	40	Culturally appropriate health information
	43	Building skills to speak up and ask questions at health care visits
	49	Information about risk of getting cancer again in the future (if in remission)
	50	Talking with relatives and doctors about family health history
	51	Information on ways to reduce risks of getting cancer
	52	Information on who should get cancer screening and when
	54	Information on how to use insurance benefits (examples: free preventive care, copays, allowed health care providers)
	55	Partnering with community organizations to share health information (examples: schools, faith-based, employers, local leaders)
	58	Use of multiple media sources for health information (examples: mail, flyers, advertisements, websites, social media)
3. Equitable accessibility	3	Access to places with affordable healthy foods (examples: grocery stores, farmers markets)
	5	Access and availability of high-speed internet
	6	Distance to hospitals or clinics
	11	Quality or trust of local health care facilities
	13	Access to at-home cancer screening (examples: Cologuard, fecal sample tests)
	14	Access to genetic screening and testing
	15	Access to needed doctors and specialists
	16	Availability of community health workers
	17	Bringing cancer screening to local communities (examples: mobile units, more screening locations)
	18	Out-of-pocket health care costs (examples: medications, treatment procedures)
	19	Expanded access to Medicaid
	20	Insurance coverage of pre-existing conditions
	21	Telehealth options for health care services
	22	Access to affordable nicotine replacement products (examples: patches, gum, lozenges)
	26	Help for traveling to cancer treatment (examples: car, gas, place to stay, someone to drive you)
	27	Access to hospice or comfort care
	30	Clear communication between health care providers and patients
	31	Communication across multiple doctors about a patient’s care
	32	Getting second or third opinions of treatment options
	33	Established relationship and trust with a health care provider
	34	General trust or confidence in health care
	37	Access to diverse/minority health care providers
	38	Bilingual staff or interpreters
	41	Health information at low reading levels
4. Perceptions, beliefs, and stigmas	7	Pollution in water, air, or soil that can cause cancer
	8	Stigma surrounding cancer
	10	Other priority health issues in the community besides cancer (example: addiction, diabetes)
	12	Stigma around mental health
	36	Vaping (examples: Juul, e-cigarettes)
	39	Cultural beliefs about seeking health care (examples: rural/Appalachian, immigrants, African American, LGBTQ+)
	45	Embarrassment or privacy concerns about cancer diagnosis
	46	Belief that changing behavior won’t make a difference (examples: smoking, nutrition, exercise)
	48	Fear or avoiding cancer screenings (examples: out-of-sight/out-of-mind mentality)
	53	Myths around cancer treatments and chances of surviving cancer
5. “Kentucky Uglies”: Current status of cancer and risk factors	9	Community ties to local industry (examples: tobacco, mining, farming, factories)
	63	Cancer rates related to tobacco are higher than in the US overall (some of the worst rates of lung, head/neck, kidney, and bladder cancers)
	64	Cancers rates related to obesity are higher than in the US overall (some of the worst rates of colorectal, pancreatic, and brain cancers)
	65	Cancers rates related to HPV are higher than in the US overall (some of the worst rates of cervical and head/neck cancers)
	66	New hepatitis C virus infection rate is among the highest in the US (linked with opioid injection use/a known cause of liver cancer)
	67	Some cancers linked to environmental exposures are more common than in the US overall (examples: lung, kidney, melanoma, leukemia, bladder)
	70	Higher cancer death rates in counties with lower education versus higher education
	72	About 1 in 4 adults are current smokers, with higher rates in Appalachia (second-highest rate in the US)
	73	1 in 5 youth and 2 in 5 adults are obese (among the highest rates in the US)
	74	1 in 3 adults fail to get any physical activity outside of work (third-worst in the US)
6. Disadvantages in Appalachian, Black, and Hispanic communities	47	Hassle/unpleasantness of cancer screening
	59	Breast cancer screening rates are similar to rate in US overall but still need improvement
	60	Lower colorectal cancer screening rates in Appalachia
	61	Lower cervical cancer screening rates in rural areas and Appalachia
	62	Lung cancer screening rates are low (even worse in the Black community)
	68	More people die from certain cancers in rural and Appalachian areas than the US overall (examples: lung, colorectal, kidney, leukemia)
	69	More Black Kentuckians die from certain cancers than White Kentuckians (examples: uterine, prostate, stomach, myeloma)
	71	Only about half of youth are fully vaccinated against HPV (even less in rural areas)
	75	Higher levels of poverty among rural, Appalachian, Black, and Hispanic Kentuckians than among the US population
	76	One-third of counties have more than 20% of people living in persistent poverty since 1980 (mostly in rural Appalachia)
	77	Fewer adults have a college degree than in the US overall (even lower among rural, Appalachian, Black, and Hispanic Kentuckians)
	78	Math and reading proficiency scores among K-12 public school students are often lower than the US average
	79	Workers are more likely than workers in the US overall to hold jobs at or below minimum wage, especially women
	80	1 in 4 Hispanic Kentuckians have no health insurance (otherwise Kentucky’s uninsured rates are better than US rates)

Abbreviations: HPV, human papillomavirus; LGBTQ+, lesbian, gay, bisexual, transgender, queer.
